# Pre-treatment oral microbiome analysis and salivary Stephan curve kinetics in white spot lesion development in orthodontic patients wearing fixed appliances. A pilot study

**DOI:** 10.1186/s12903-023-02917-z

**Published:** 2023-04-24

**Authors:** Raisa Queiroz Catunda, Khaled Altabtbaei, Carlos Flores-Mir, Maria Febbraio

**Affiliations:** 1 School of Dentistry, University of Alberta, Edmonton, AB T6G 2E1 Canada; 2 Division of Periodontology, School of Dentistry, University of Alberta, Edmonton, AB T6G 2E1 Canada; 3 Division of Orthodontics School of Dentistry, University of Alberta, Edmonton, AB T6G 2E1 Canada; 4 Division of Oral Biology, School of Dentistry, University of Alberta, 11361-87th Avenue, Edmonton, AB T6G 2E1 Canada

**Keywords:** White spot lesion, Microbiome, Saliva pH

## Abstract

**Background:**

White spot lesions (WSLs) are a formidable challenge during orthodontic treatment, affecting patients regardless of oral hygiene. Multifactorial in nature, amongst potential contributors to their development are the microbiome and salivary pH. The aim of our pilot study is to determine if pre-treatment differences in salivary Stephan curve kinetics and salivary microbiome features correlate with WSL development in orthodontic patients with fixed appliances. We hypothesize that non-oral hygiene determined differences in saliva could be predictive of WSL formation in this patient population through analysis of salivary Stephan curve kinetics, and that these differences would further manifest as changes in the oral microbiome.

**Methods:**

In this prospective cohort study, twenty patients with initial simplified oral hygiene index scores of “good” that were planning to undergo orthodontic treatment with self-ligating fixed appliances for at least 12 months were enrolled. At pre-treatment stage, saliva was collected for microbiome analysis, and at 15-minute intervals after a sucrose rinse over 45 min for Stephan curve kinetics.

**Results:**

50% of patients developed a mean 5.7 (SEM: 1.2) WSLs. There were no differences in saliva microbiome species richness, Shannon alpha diversity or beta diversity between the groups. *Capnocytophaga sputigena* exclusively and *Prevotella melaninogenica* predominantly were found in WSL patients, while *Streptococcus australis* was negatively correlated with WSL development. *Streptococcus mitis* and *Streptococcus anginosus* were primarily present in healthy patients. There was no evidence to support the primary hypothesis.

**Conclusions:**

While there were no differences in salivary pH or restitution kinetics following a sucrose challenge and no global microbial differences in WSL developers, our data showed change in salivary pH at 5 min associated with an abundance of acid-producing bacteria in saliva. The results suggest salivary pH modulation as a management strategy to inhibit the abundance of caries initiators. Our study may have uncovered the earliest predecessors to WSL/caries development.

**Supplementary Information:**

The online version contains supplementary material available at 10.1186/s12903-023-02917-z.

## Background

The formation of white spot lesions (WSLs) during orthodontic treatment is an aesthetic and morphological adverse effect caused by plaque-mediated demineralization of the tooth surface around brackets. They are associated with changes in enamel morphology and can lead to dissatisfaction with tooth surface appearance [1, 2]. The incidence and prevalence of WSL development during orthodontic treatment vary between 23.4 and 72.9% and 30-75.6%, respectively, depending on the assessed parameters (age, diagnosis criteria, initial caries assessment). Incidence is generally assessed throughout orthodontic treatment (18–24 months on average), and for most studies that included prevalence, patients were at least 12 years old [3–14].

Although the expectation is that WSL occurrence in orthodontic patients results from inadequate oral hygiene, Geiger et al. showed that even when oral hygiene compliance was moderate to excellent, 42% and 15% of such patients, respectively, still developed WSLs [4]. Another study found that patients with good or moderate oral hygiene still had a prevalence of WSLs ranging from 23 to 68%, with an average development of 1 new WSL for patients that had good oral hygiene, 1.4 WSLs for patients with moderate oral hygiene and 3.3 WSLs for patients with poor oral hygiene (follow-up 9 to 25 months) [15]. These studies suggest that WSL formation is multifactorial and is not solely dependent on competency in performing oral hygiene.

While WSLs share the same demineralization process as caries, their clinical presentation and lack of cavitation are more considered an aesthetic compromise and a precursor to frank dental caries. Fixed orthodontic appliances render oral hygiene more challenging, which, similar to interproximal spaces, favors the creation of new habitats for biofilm accumulation, leading to loss of enamel hydroxyapatite and, finally, the clinical appearance of white spots. A recent study of the microbiota in different types of carious lesions, from WSLs to dentin caries, showed that *Streptococcus mutans*, considered to be highly associated with caries, were low in abundance in WSLs, comprising only 0.73% of the total bacterial community [16, 17]. Interestingly, WSLs had lower richness and diversity than open dentin cavities. *Streptococcus*, *Rothia*, *Leptotrichia* and *Veillonella* were found at higher levels in carious enamel lesions, whereas *Lactobacillus*, *Shlegelella*, *Pseudoramibacter* and *Atopobium* were associated with dentin lesions. This supports the etiologic contribution of non-mutans bacterial species and their roles in the spectrum of the development of caries [18–21].

The essential association between pH and caries formation was illustrated by the work of Stephan in 1944 [22]. In his classic clinical trial, he showed that a sucrose mouth rinse led to a drop in pH in dental plaque, followed by gradual restoration to baseline over time (“Stephan curve”). The three phases of the Stephan curve are (1) rapid drop in pH, due to fermentation of sucrose by acid-producing bacteria; (2) demineralization of enamel if the pH drop is below 5.5; (3) gradual increase back to baseline within 30 to 60 min. In Stephan’s study, patients could be classified into caries-free to high caries activity groups based on the association between pH drop and the development of lesions over 12 months [22]. The same pattern of acidification and neutralization occurs in saliva. Saliva has buffering properties that typically act to prevent caries; if the acid neutralization effect of saliva is diminished, this may lead to a higher prevalence of WSLs [23]. In addition, reduced buffering capacity could lead to prolonged low salivary pH, which would effectively select for acidogenic and aciduric bacteria, potentially resulting in saliva acting as a microbial seeding reservoir to accumulating plaque around orthodontic brackets [24]. Saliva buffering capacity is independent of oral hygiene and most likely genetically determined in healthy individuals.

Most orthodontic WSL studies have focused on one aspect of the multifactorial causation, e.g. specific bacterial species, rather than the microbiome as a whole. They have not considered the dynamic protective effects of saliva [3, 6, 10–13, 25, 26]. As such, our understanding of the association between inherent saliva buffering differences in healthy individuals and the formation of WSLs around orthodontic brackets is incomplete. The primary objective of this prospective cohort study is to determine if pre-treatment salivary Stephan curve kinetics are associated with WSL development, with a secondary objective of investigating the contribution of the salivary microbiome at baseline on Stephan curve kinetics and the development of WSLs.

## Methods

### Study design, inclusion and exclusion criteria

The number of patients to enroll in this pilot study in order to have 2 equal groups for control and WSL cases was calculated at 80% power with a confidence level of 95% (precision of at least 5% is recommended when expected prevalence ranges between 10 and 90%) using the following website: https://clincalc.com/stats/samplesize.aspx [9]. Previous experience in the orthodontic clinic at the University of Alberta showed that 60% of patients developed some signs of a WSL (Flores-Mir, C., unpublished observations). Using this estimation and the selection of two independent study groups with dichotomous endpoint, we calculated that the number of patients was 16 (8 per group). Due to the study length, to allow for possible drop-out of 20%, we set a recruitment target of 10 patients per group, for a total of 20 patients.

Study participants were recruited according to a protocol approved by the University of Alberta Health Research Ethics Board (Pro00099341). Written informed consent or assent was obtained from all participants. Patients were assessed for good oral health before appliance placement based on the simplified oral hygiene index (OHI-S, Green and Vermillion, 1964) prior to start of treatment and every three months [27]. Briefly, six teeth were scored for their debris and calculus indexes (Fédération Dentaire Internationale (FDI) notation: #16: upper right 1st molar, #11: right upper central incisor, #26: upper left 1st molar, #36: lower left 1st molar, #31: left lower central incisor, #46: lower right 1st molar). Debris was scored 0–3 as follows: 0: no debris, 1: soft debris < 1/3 of tooth surface, 2: soft debris > 1/3 and < 2/3 of tooth surface, and 3: debris covering > 2/3 of tooth surfaces. A similar scoring was done for calculus based on crown coverage. The sum for each index was divided by the number of teeth examined (6) and then added together for the total OHI-S index score. The scores related to oral health are as follows: 0.1–1.2: good; 1.3–3.4: fair; 3.1-6.0: poor [10]. Patients with OHI-S scores between 0.1 and 1.2 (good) that had fully erupted second molars, and a treatment plan for fixed self-ligating orthodontic appliances (at least from 1st molars to 1st molars) for a minimum of 12 months duration, were recruited. There were no restrictions pertaining to age, sex or type of malocclusion. Patients who had any intra-oral appliances other than fixed self-ligating brackets, had any surgery planned during treatment or WSLs of any origin at the beginning of treatment were excluded. Patients that had systemic disease, were smokers or on medication were also excluded. Those enrolled had their hygiene appointments within three months prior to bracket placement. Patients were advised to brush twice a day with a soft-bristled toothbrush and fluoride toothpaste (1450 ppm) and floss daily. All patients also received standard dietary advice, such as to avoid hard/sticky/crunchy foods and sugary drinks. Patients that did not maintain an OHI-S 0.1–1.2 score throughout the treatment were also excluded.

### WSL assessment

WSLs were assessed according to the modified WSL index (Gorelick et al., 1982) by evaluating the buccal surface of individual teeth for presence or absence and severity [6]. Scores were noted at treatment start (all = 0) and every three months until month 12. The assessment was performed under direct illumination using a dental chair light after drying the teeth with compressed air for 5 s.

Two orthodontic residents, in a blinded manner, independently used direct visualization to assess for WSLs. Photographs were taken on the same day of all teeth, regardless of whether a WSL had been detected. Later on, the same residents were asked to assess the photographs for WSLs and a third one (the lead author) would match their clinical assessment with their assessment via photographs. Thus, WSLs were assessed by two methods, in person at the time of appointment and subsequently, using photographs taken at the time of appointment. For the photographs, patients were asked to sit upright in their habitual occlusion and with relaxed lips and mentalis muscles. Their heads were positioned in a way that the Frankfurt horizontal plane was parallel to the ground. In this way, the residents were able to align the midsagittal plane of the patient’s head with the middle of the camera lens. There were a total of 5 pictures taken: right buccal, left buccal, frontal, upper and lower occlusal shots. A metal retractor was used to completely expose teeth (up to second molars) and double-sided intraoral mouth mirrors were also used. Photographs were taken with a Canon EOS Rebel T7 18-55 mm camera/lens (cat# EOSREBELT7KITDC). Images were magnified and assessed independently by the 2 residents. If there was disagreement, a discussion ensued. If there was still disagreement, the procedure called for consultation with a third resident.

### Saliva collection and salivary Stephan curve kinetics

Stephan curve kinetics were determined before the placement of fixed appliances. Patients were asked to refrain from eating, drinking or chewing gum for 30 min. Patients rinsed with water for 30 s, and an initial ~ 1 ml sample of saliva was obtained for pH analysis and ~ 2 ml for microbiome analysis. Next, patients rinsed with a 10% sucrose solution for 30 s, and saliva samples were collected after 5, 15, 30 and 45 min. pH was determined using a microelectrode (Cole Parmer pH meter PH6+, Quebec, QC).

### DNA isolation and sequencing

Immediately after obtaining the saliva samples, the steps recommended by the manufacturer of the kit used were followed to preserve the DNA (Microbiome DNA Isolation Kit, Norgen Biotek Corp, Thorold, ON). For 16s rDNA amplicon sequencing, primers for two sets of variable regions were used, as one or the other may, in some cases, more effectively identify genera, and this allowed for the retrieval of a broader microbiome spectrum than what would be achievable with one primer set. The V1-3 region was sequenced using 27 F and FwR1 (ACACTCTTTCCCTACACGACGCTCTTCCGATCTGAAKRGTTYGATYNTGGCTCAG) and (GTGACTGGAGTTCAGACGTGTGCTCTTCCGATCTACGTNTBACCGCDGCTGCTG). The V4-5 region was sequenced using the following primers: Forward: 515FP4-FwR1, 515FP3-FwR1, 515FP2-FwR1, 515FP1-FwR1 (ACACTCTTTCCCTACACGACGCTCTTCCGATCTCAAGTGCCAGCMGCCGCGGTAA, ACACTCTTTCCCTACACGACGCTCTTCCGATCTACGTGCCAGCMGCCGCGGTAA, ACACTCTTTCCCTACACGACGCTCTTCCGATCTTGTGCCAGCMGCCGCGGTAA, ACACTCTTTCCCTACACGACGCTCTTCCGATCTGTGCCAGCMGCCGCGGTAA) and reverse: 806RP4-RvR2, 806RP3-RvR2, 806RP2-RvR2, 806RP1-RvR2 (GTGACTGGAGTTCAGACGTGTGCTCTTCCGATCTCATGGACTACHVGGGTWTCTAAT, GTGACTGGAGTTCAGACGTGTGCTCTTCCGATCTACGGACTACHVGGGTWTCTAAT, GTGACTGGAGTTCAGACGTGTGCTCTTCCGATCTTGGACTACHVGGGTWTCTAAT, GTGACTGGAGTTCAGACGTGTGCTCTTCCGATCTGGACTACHVGGGTWTCTAAT). Sequencing was performed on the Illumina MiSeq platform. Adaptors were removed using CutAdapt [28]. Parameters for trimming and overlap needed for merging were determined with Figaro [29]. Merged sequences were algorithmically corrected to produce Amplicon Sequencing Variants (ASVs) using software DADA2 [30]. DADA2 was used to bin the nucleotide-corrected ASVs to their identifying taxa using the naive Bayesian classifier against a SILVA rRNA database (v138.1, provided by DADA developer here: https://zenodo.org/record/4587955#.Ykc0By971jc) using the assignTaxonomy command (Supplementary files 1 and 2) [30]. Next, each ASV was condensed based on its taxonomy to yield a table where each taxa is represented only once (phylotoast reference: 10.1038/srep29123)]. Analysis was done at this taxonomy level. To avoid overestimation, primer averaging was done [31].

### Statistical analysis

Data were entered into Excel (version 2208, Microsoft), and statistical calculations were performed in IBM Statistical Package for Social Sciences (SPSS, version 28). For salivary Stephan curve analysis, the mean and standard error of the mean (SEM) of pH were calculated for each assessment time and were stratified between the case and control groups. To account for clustering of teeth within the mouth, in the analysis the data were dichotomised by participant into ‘Yes, the participant had at least one new WSL’, or ‘No the participant did not have at least one new WSL’. In this way, a patient with one WSL was analyzed in the same way as a patient with more than one. Data normality was verified by the Kolmogorov-Smirnov test. The alpha level to determine significance was 5%. Repeated-measures ANOVA was performed to compare pH at the different Stephan curve time points with occurrence of WSLs, using Bonferroni correction for multiple comparisons. This analysis compares pH at different time points amongst WSL or no WSL patients (these are the repeated observations). The measurements were taken once, prior to brackets placement, for each patient at each time point. For microbiome analysis, alpha and beta-diversity were interrogated using an automated pipeline (Forays into Automating Laborious Analysis of Phylogeny (FALAPhyl): https://github.com/khalidtab/FALAPhyl). Feature-abundance testing was examined via Linear discriminant analysis (LDA) Effect Size (LefSe) [32].

## Results

**Fig. 1:**
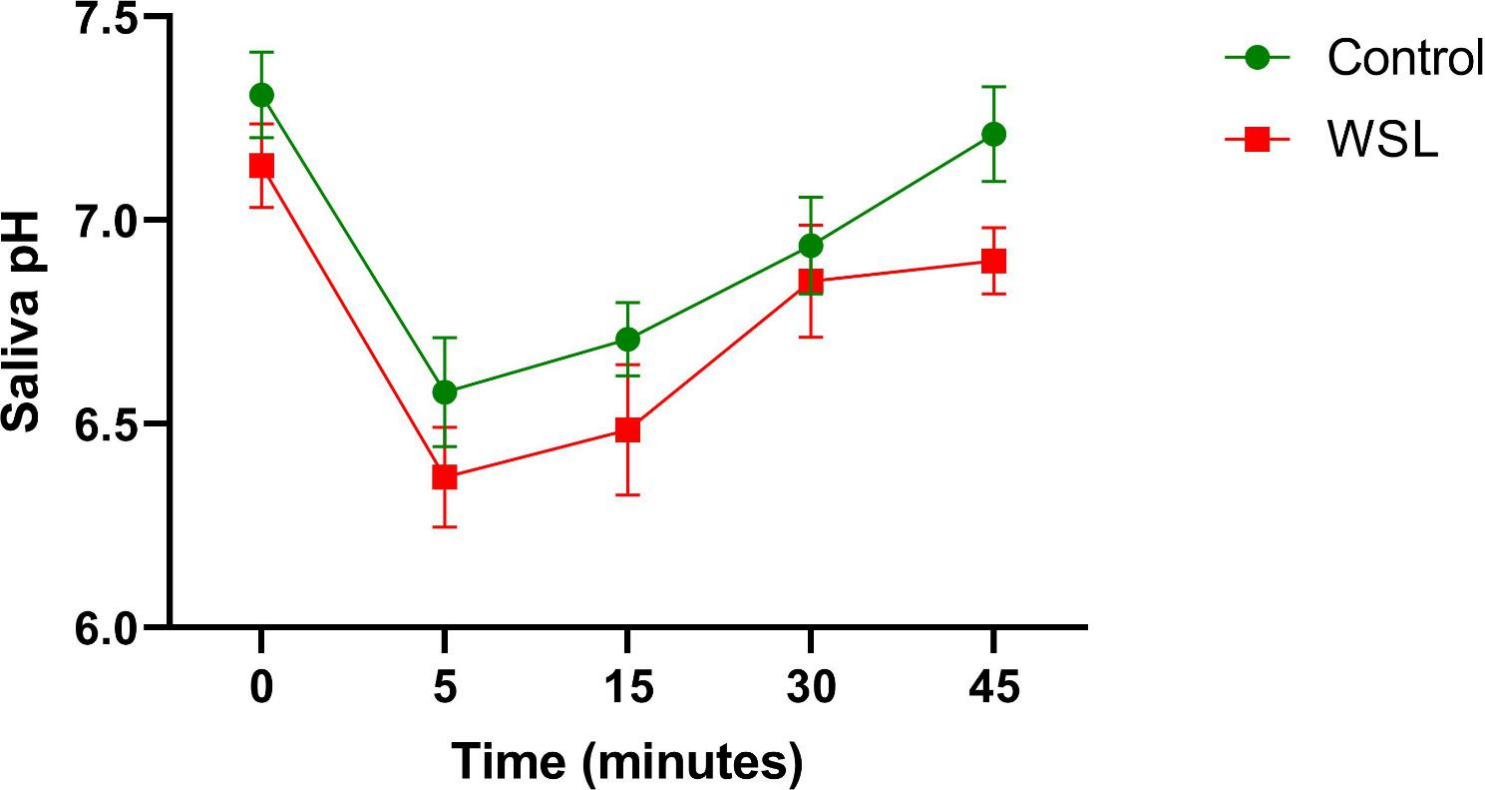
Pre-treatment Stephan curves for those with and without WSL at 12 months

Table 1 shows the descriptive statistics of the patients included in this study. The increase in age in the control group was driven by 3 patients that were above 40 years of age. The mean age and SEM of the other 7 controls was 14 + 1. Figure 1 shows the aggregate Stephan curves of the WSL group versus control; there were no statistically significant differences at any time points (p > 0.05, repeated measures ANOVA). In the control group, a comparison of pH at each data point between those greater than 40 years of age with those less than 40 years of age also showed no significant differences. While there was no evidence to support the primary hypothesis, it is possible that significant differences in initial Stephan curve kinetics could be uncovered using this protocol with a greater sample size. Thus, this can be considered a pilot study for the calculation of such sample size.

Sample size would be dependent on which parameter being studied. In each case following, we calculated sample size at 80% power with alpha ≤ 0.05. Based on our data, at time 0, the sample size would need to be 209/group to detect a difference. At time 5 min, the sample size would need to be 59/group; at time 15 min, the sample size would need to be 51/group; at time 30 min, the sample size would need to be 343/group and at time 45 min, the sample size would need to be 17/group. For area under the curve, the sample size would need to be 46/group. For maximum pH drop, the sample size would need to be 4808/group. For drop in pH at 5 min, the sample size would need to be 118/group. For difference at 45 min, the sample size would need to be 73/group. However, we do not consider increasing the sample size to be the best approach to take for future studies. We would instead suggest changing the study design as outlined in the discussion.

**Table 1:** Results from the analysis of variance for the representative terms of the data and collinearity

	Age (SEM)	Male/female ratio	WSL count (SEM)	Sample size
**Control**	23.0 (4.6)	1:4	0	10
**WSL**	13.1 (0.4)	1:1	5.7 (1.2)	10

Alpha diversity rarefaction curves of the observed taxa showed that we had adequate sequencing depth (Supplementary Fig. 1). In terms of the saliva microbiome analyses, there were no global differences between the two groups in terms of Chao-1 index, a measure of alpha diversity or species richness (p > 0.05, Mann–Whitney, Supplementary Fig. 2A, B), Shannon diversity index (p > 0.05, Mann–Whitney), or beta diversity, a measure of the similarity between the groups (p > 0.05, ADONIS of Phylogenetic Isometric Log-Ratio (PhILR) distances, Supplementary Fig. 2C).

Canonical correspondence analysis was performed on the log-transformed rarefied taxa counts to assess the associations between the different terms and species using two models. The first model used raw pH terms and did not result in significant associations (p > 0.05). The second model used pH as a function of restitution to baseline. The restitution of pH was coded as delta pH (ΔpH) between the desired timepoint compared to the initial (pH_initial_ - pH_x_) where x = time in minutes (e.g., ΔpH_5_, where x = 5 min). Therefore, the drop from initial pH was represented as a positive number; higher positive numbers represented a greater difference from baseline pH. The second model resulted in ΔpH_5_ being the best representative of the explanatory variables for the log transformed taxa counts, as shown in Table 2.

**Table 2:** Results from the analysis of variance for the representative terms of the data and collinearity

Variables	Degrees of freedom	Chi-Square	F	P-value
ΔpH_5_	1	0.04	2	> 0.01
Residual	18	0.4		

P < 0.05: statistically significant

**Fig. 2:**
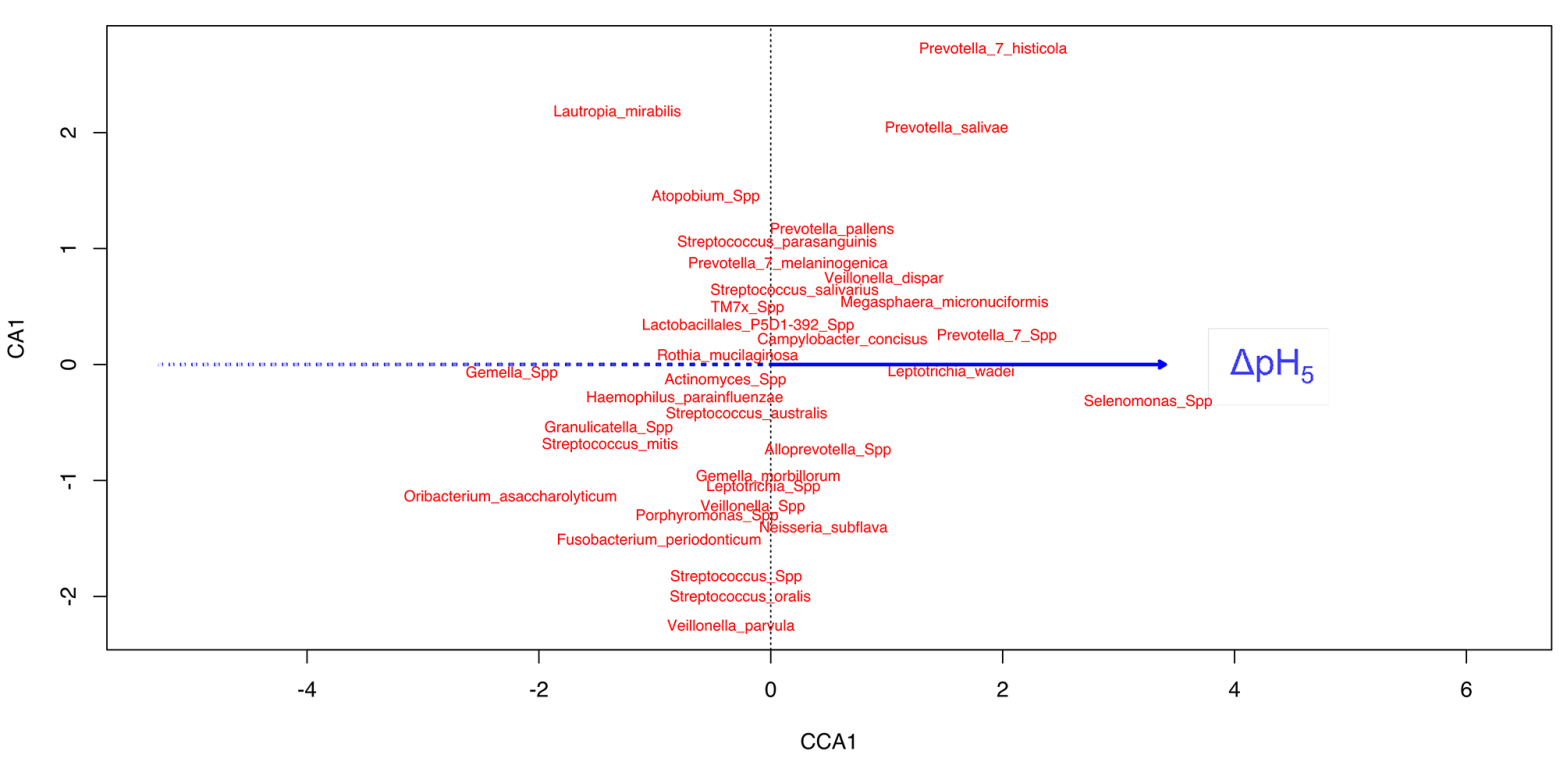
Bimodal relationship between species abundances: the linear gradient of the statistically significant continuous representative term of the data, ΔpH_5_ (blue line). The solid line represents a positive increase in the term, while the dashed line represents a decrease in the term. The placement of species/genera (points) is indicative of their association to the continuous term, when an imaginary-line originating from the continuous variable at a right-angle (the solid, or dashed line) intersects with that point. Intersections at the peripheries, as opposed to those closer to the middle, indicate increased strength of association

Constraining (canonical) the model reduced the inertia from 0.35 to 0.038; as such, these variables were responsible for 10.9% of the inertia/variance within the model. The unimodal effect of the terms on species abundances was graphically interrogated (Fig. 2). Early pH restitution (ΔpH_5_) affected species abundances in agonistic and antagonistic fashions.

**Fig. 3:**
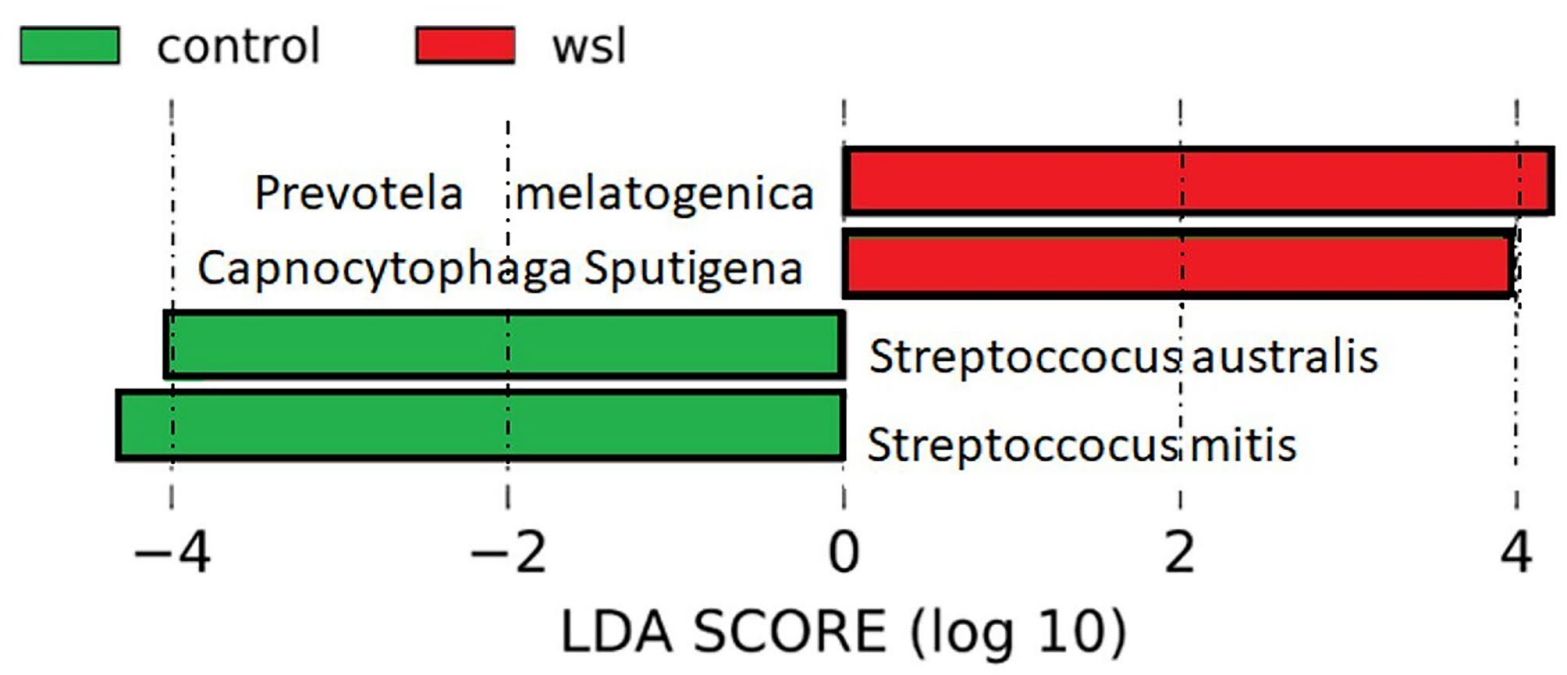
Visualization of differential features ranked by effect size

The LEfSe method was used to support high-dimensional class comparisons of the taxa (Fig. 3). *Capnocytophaga sputigena*, a gram-negative acid-producing bacterium, was exclusively found in WSL patients. Interestingly, *Prevotella melaninogenica*, a pathogen primarily associated with periodontal disease, was also mostly observed in WSL patients. Bacteria such as *Streptococcus mitis* and *Streptococcus australis* were primarily present in healthy patients. *Streptococcus mitis* has been reported as one of the least likely bacteria to contribute to caries, whereas *Streptococcus australis* is considered part of the *Streptococcus mitis* group and is known for arginine hydrolysis and production of alkaline phosphatase [33].

## Discussion

Caries result from a dynamic process in which a dysbiotic biofilm facilitates the demineralization of tooth enamel through the production of acid by the microbiota [34]. WSLs are the first clinical indication of this process, hence the importance of understanding initiating and possible inhibiting factors. The increase in WSLs during orthodontic treatment is a significant concern to clinicians, given that even patients with excellent to good oral hygiene still develop some degree of demineralization during treatment [15]. In this study, we compared two groups of patients treated at the same time in a graduate orthodontic clinic: those that did or did not develop WSLs, in accordance with the inclusion/exclusion criteria outlined in the methods section of this paper. Our hypothesis was that non-oral hygiene determined differences in saliva, prior to bracket placement, could be predictive of WSL formation in this patient population, through analysis of salivary Stephan curve kinetics, and that these differences would further manifest as changes in the oral microbiome.

In the WSL group, 5.7 (SEM: 1.2) WSLs per patient (range 1–12 WSLs) developed. This was despite efforts to minimize WSL formation through patient selection and appliance type. We only enrolled patients with good oral hygiene (OHI-S scores between 0.1 and 1.2), and this was maintained throughout the study length for patients to be included at the end. Because not all orthodontic devices/approaches (e.g. self-ligating brackets, removable appliances) have the same predisposition to affect oral hygiene, we only included patients with a treatment plan that used archwires with self-ligating brackets and excluded those with elastomeric rings, since they make cleaning more difficult [36–40]. In this way, our study was designed to capture inherent differences in saliva in an orthodontic setting in a patient pool in which oral hygiene should be well maintained. In line with what has been reported in the literature and previous experience in our clinic, however, ~ 50% of our patients still developed WSLs [35]. This finding alone indicates a need for greater research into the factors that contribute to the development of these lesions.

In our study, pre-treatment salivary pH alone could not predict WSL occurrence in orthodontic patients. Salivary Stephan curve kinetics showed no statistically significant differences between the groups across the different time points. Stephan originally performed measurements in plaque, but as in our study, others have focused on salivary pH and pH recovery [22, 36–39]. Salivary pH is a major factor controlling plaque pH [40]. In studies measuring salivary pH in healthy, caries-free/inactive individuals, baseline values ranged from 6.8 to 7.6, similar to our findings [37, 41]. The mean lowest pH for subjects that developed WSLs was ~ 6.4, while the mean lowest pH for those that did not was ~ 6.6. These values are well above the critical pH for demineralization of enamel (5.5). In addition to the dilution effect, saliva contains 3 different buffering systems. In a patient pool such as ours (ORI-S 0.1–1.2), we would not expect saliva pH to drop below 5.5. However, a drop of 0.2 in saliva could reflect a clinically relevant drop in pH at a specific tooth surface.

Classical paradigms investigate the effect of pH over time on various tooth structures as related to demineralization properties and microbiome changes compared to a baseline state as a result of some perturbation. In this study, we correlated salivary Stephan curve kinetics with pre-treatment patient microbiomes to try to define an at-risk population for WSL formation. Indeed, while the raw pH changes were not significantly associated with any unimodal microbial abundance changes, pH drop at 5 min was. This suggests that the severity of pH change (at 5 min) from baseline was significantly associated with specific bacterial species abundances, suggesting that saliva, as an ecological environment, is influenced by such pH perturbation. These results do not show causality, however.

*Selenomonas spp* were related to an increase in ΔpH_5_ (more severe pH drop). *Selenomonas* are gram-negative, motile bacteria found in the gastrointestinal tract and oral cavity in biofilms. They contribute to plaque structure, produce acetic, lactic and propionic acids as metabolic products, and this may account for their association with increased ΔpH_5_ [42]. They produce acid and thrive in acidic environments [43–46]. Previous reports suggested an association between dentinal caries and subgingival plaque [42, 47, 48]. It is tempting to speculate that these bacteria seed and initiate the pH shift in the biofilm that creates the environment for pathogenic bacteria associated with caries and thus may be essential predecessors.

*Gemella spp* are gram-positive cocci found on mucous membranes of the oral cavity and upper respiratory tract; they have also been isolated from human dental plaque. In our study, they were negatively associated with ΔpH_5_. *Gemella spp* have been associated with oral health in children and young adults [47]. Another study; however, found that *Gemella sanguinis* and *Gemella haemolysans* were associated with gingivitis in an adolescent orthodontic patient population, and a recent metagenomic analysis included *Selenomonas spp* and *Gemella spp* as co-prevalent with *Streptococcus*, *Veillonella* and *Actinomyces* in the saliva of patients with caries [49, 50]. *Gemella spp* ferment glucose, sucrose and sugar alcohols to yield acid in anaerobic and aerobic conditions [51]. Similar to *Selenomonas spp*, they are adapted to an acidic environment.

LEfSe analysis showed that *Capnocytophaga sputigena* was exclusive to WSL patients. This differed from the findings of Tanner et al. Generally considered oral commensals, *Capnocytophaga spp* require CO_2_ and ferment carbohydrates to succinate and acetate. They have been associated with gingivitis, periodontal disease, halitosis, diabetes and pre-diabetes [52–55]. Their relationship with caries remains equivocal; one study found *Capnocytophaga spp* to be associated with caries-active individuals, while several suggest they are more often a sign of a caries-free state [52–54]. In a study of microbial succession in biofilms following professional cleaning, while no differences in bacterial species colonization were uncovered between healthy and periodontitis patients, *Capnocytophaga sputigena* was found in subgingival biofilms seven days after initial colonization by *Streptococcus mitis, Veillonella parvula* and *Capnocytophaga gingivalis* [55]. *Prevotella melaninogenica*, another pathogen that is commonly associated with periodontal disease, advanced carious lesions and active-caries saliva, was found mostly in WSL patients in our study [56–59]. Together with the presence of *Capnocytophaga sputigena*, these results suggest a more mature plaque environment reflected in the saliva of WSL developers. This is interesting because these parameters were assessed more than two months before the first WSL developed in one of our patients.

*Streptococcus mitis* and *Streptococcus australis* were primarily present in healthy patients. *Streptococcus mitis* was reported to be one of the least likely bacteria to contribute to caries. After the introduction of 16s sequencing, this species could be differentiated from *Streptococcus mutans* and shown to be one of the least resistant to low pH when compared to other *Streptococcus* species [60]. *Streptococcus australis* can hydrolyse arginine to ammonia, a base that can neutralize acid and plays a key role in plaque homeostasis by inhibiting the growth of aciduric bacteria [61]. In this way, *Streptococcus australis* antagonizes dental caries pathogenesis. This species can produce alkaline phosphatase, which increases the calcium and phosphate content of saliva and plaque, promoting remineralization [33, 62].

Overall, our data indicate an increased abundance of acid-producing bacteria in the saliva of WSL developers, but interestingly, not the usual suspects, *Streptococcus mutans Streptococcus sobrinus*, and *Lactobacillus acidophilus*, which are considered to be the major caries initiators. Our data also differ from others that researched the microbiota of WSLs. This is likely because our microbiome analysis was done prior to WSL detection. Thus, our study may have uncovered the earliest predecessors to WSL/caries development.

### Limitations

Although we tried to standardize the patients’ bracket system, oral hygiene and enamel conditions pre-treatment, dietary habits, socio-economical background and lifestyle could not be fully controlled in this study. The control group was older compared to the case group. Extensive outlier analyses were negative and cca of the microbiome with age showed no association, thus these patients were not excluded.

It should be emphasized that at time of saliva collection and Stephan curve kinetics, all patients had just had a professional dental cleaning and all had equivalent good OHI-S scores. Collection of saliva and dental plaque are simple, non-invasive procedures that can provide a breadth of information to mechanistically address enamel demineralization and identify susceptibility in subjects. In our study, we only evaluated saliva prior to orthodontic treatment, and this parameter alone could not predict individuals more likely to develop WSLs. We did not measure dental plaque pH or plaque microbiome. These two factors can be a point for future investigation as dental plaque and saliva possess different microbial compositions and dental biofilm is known to play an important role in the progression of dental caries [18, 63, 64]. Studies in the literature point at smaller differences between microbiome plaque in orthodontic patients that developed WSLs vs. controls, however this information combined with the saliva microbiome data could potentially result in stronger associations, especially when associated with pH curves/drops [50].

Our study protocol provided no evidence that Stephan curve kinetics alone could predict occurrence of WSLs. A different study design, incorporating a higher sucrose concentration in the rinse, Stephan curve kinetics before and during orthodontics treatment and isolation of plaque for pH and microbiome analyses, may have success. Our study did find, however that certain microbiome characteristics or certain Stephan curve parameters when combined with microbiome analyses correlated significantly with the development or not of WSLs. The fact that many acid-forming bacteria were found to be related to pH restitution provides a compelling rationale for further studies.

## Conclusions

In our population of healthy subjects with initial good oral hygiene, there were no differences in pre-treatment salivary pH or restitution potential following a sucrose challenge and no global microbial differences between WSL developers and healthy patients. However, to our knowledge, this is the first time that changes in salivary pH (either absolute pH or recovery) have been found to be associated with the abundance of acid-producing bacteria in saliva and WSL development. The results suggest saliva pH modulation as a therapeutic strategy to inhibit the abundance of caries initiators. Modulation of pH could be effected by use of a rinse, a gum, a lozenge or a probiotic. The earliest predecessors to WSL/caries development may have been identified.

## Electronic supplementary material

Below is the link to the electronic supplementary material.

**Supplementary Figure 1.** Alpha rarefaction curves (observed features): depth of coverage for each patient sample (each colour represents one patient).

**Supplementary Figure 2.** Violin plots of alpha diversity representing richness and diversity: A: Chao 1 index; B: Shannon index; C. Principal coordinates analysis (PcoA) of PhILR distances between WSL patients (red) and controls (green). The ellipses represent the standard deviation of the dispersion in the two groups, with each sample connected to the centroid (larger dot) by a grey line.

Supplementary Material 3

Supplementary Material 4

## Data Availability

The dataset used and/or analyzed during the current study is available on https://www.ncbi.nlm.nih.gov/bioproject/901626.

## Abbreviations

ASV: Amplicon Sequencing Variants
FALAPhyl: Forays into Automating Laborious Analysis of Phylogeny
FDI: Fédération Dentaire Internationale
LDA: Linear discriminant analysis
LefSe: Effect Size
OHI-S: Simplified oral hygiene index
SPSS: Statistical Package for Social Sciences
WSL: White spot lesions
ΔpH: delta pH
