# Supplementary figures and images for: Pre-treatment oral microbiome analysis and salivary Stephan curve kinetics in white spot lesion development in orthodontic patients wearing fixed appliances. A pilot study
Source: BMC Oral Health. 2023 Apr 24;23:239. doi: 10.1186/s12903-023-02917-z (PMC10127078; doi:10.1186/s12903-023-02917-z)

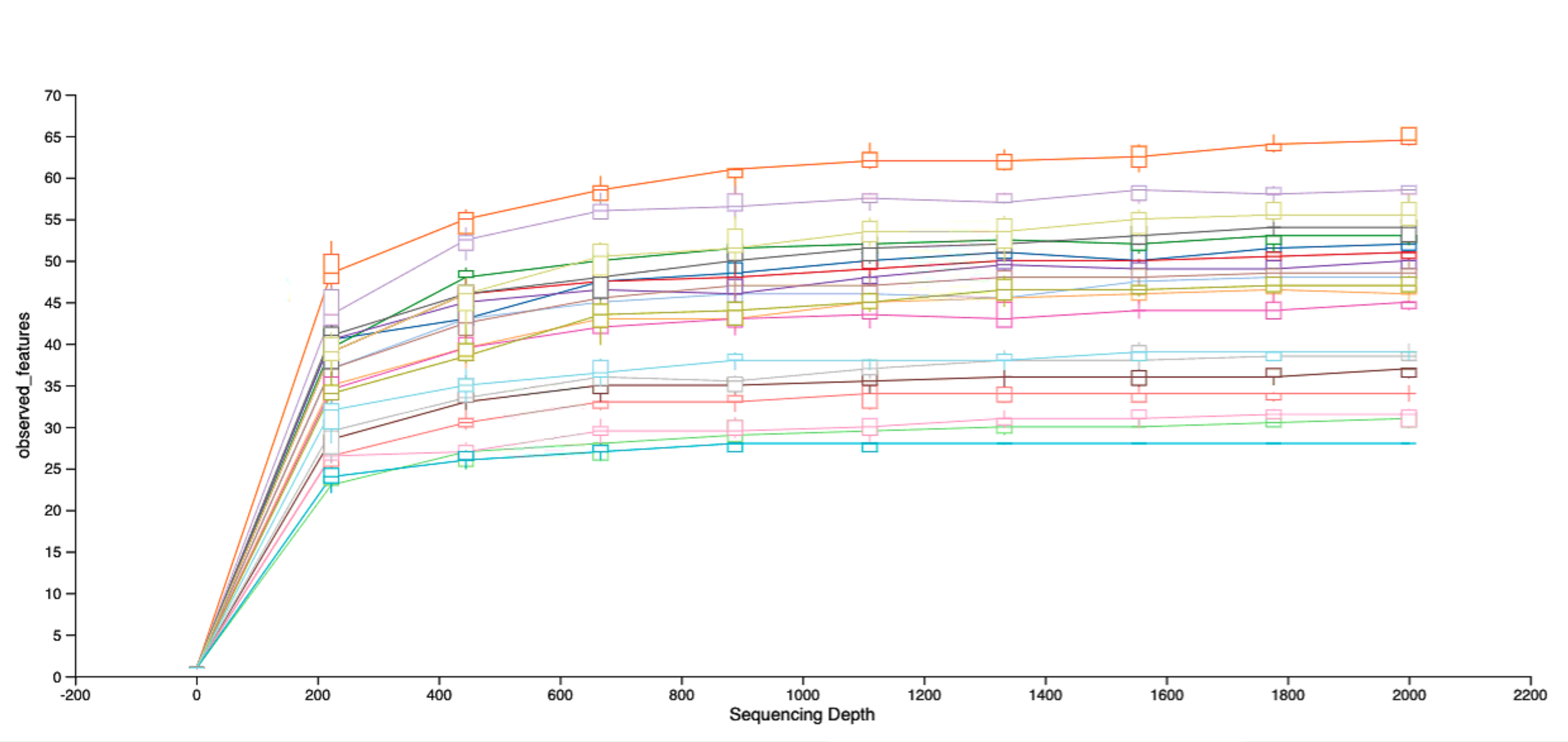

Supplement: Supplementary file 1 — Supplementary Figure 1. Alpha rarefaction curves (observed features): depth of coverage for each patient sample (each colour represents one patient). [file 12903_2023_2917_MOESM1_ESM.png]

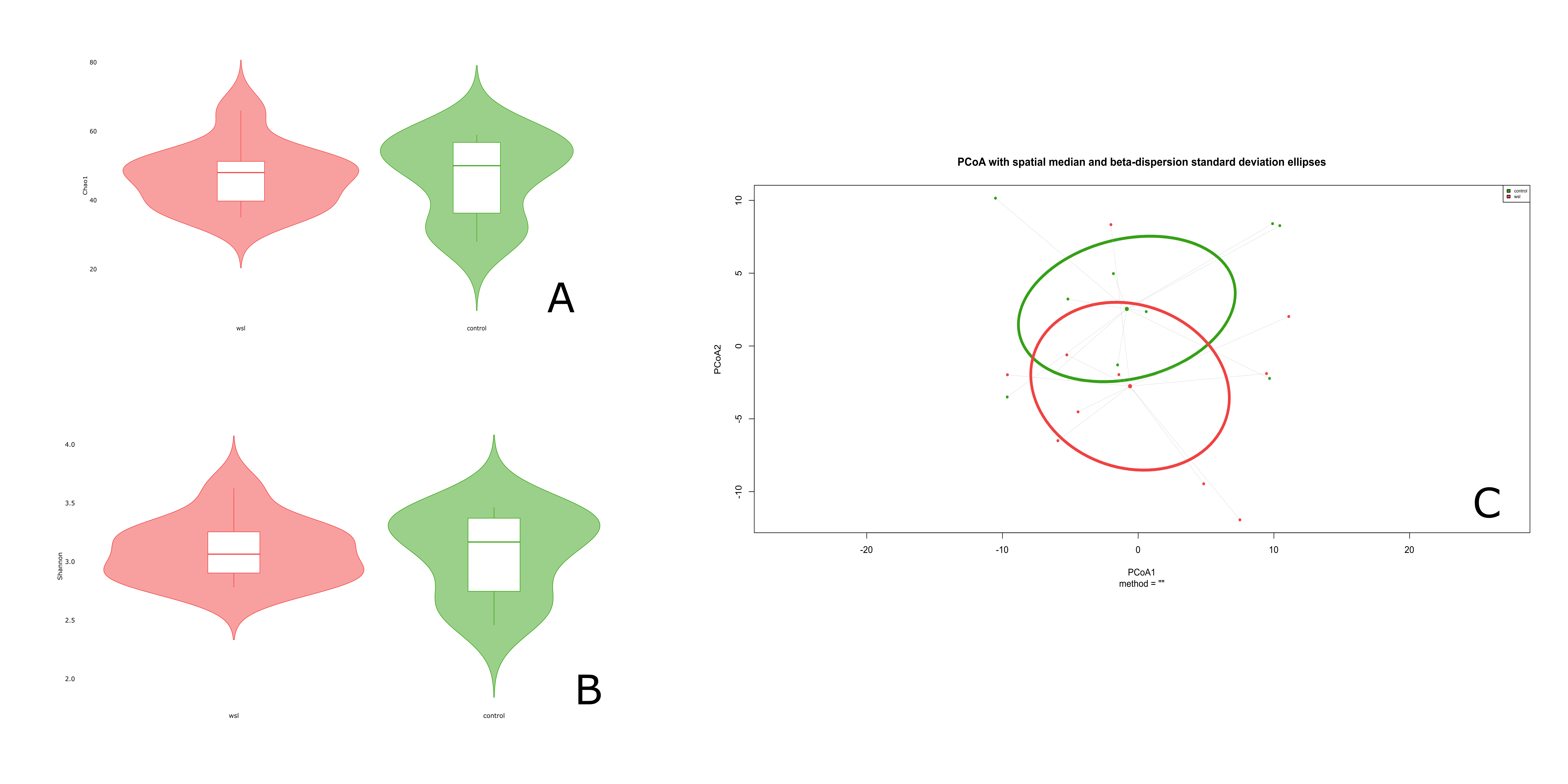

Supplement: Supplementary file 2 — Supplementary Figure 2. Violin plots of alpha diversity representing richness and diversity: A: Chao 1 index; B: Shannon index; C. Principal coordinates analysis (PcoA) of PhILR distances between WSL patients (red) and controls (green). The ellipses represent the standard deviation of the dispersion in the two groups, with each sample connected to the centroid (larger dot) by a grey line. [file 12903_2023_2917_MOESM2_ESM.png]
