# Supplementary material for: Pre-treatment oral microbiome analysis and salivary Stephan curve kinetics in white spot lesion development in orthodontic patients wearing fixed appliances. A pilot study
Source: BMC Oral Health. 2023 Apr 24;23:239. doi: 10.1186/s12903-023-02917-z (PMC10127078; doi:10.1186/s12903-023-02917-z)
Supplement: Supplementary file 3 — Supplementary Material 3 [file 12903_2023_2917_MOESM3_ESM.docx]

**Supplementary File 1: cutadap V13 and V45**

# Cutadapt for removing the adaptors

## V13

Adaptors used in this case are 27F and FwR1 (ACACTCTTTCCCTACACGACGCTCTTCCGATCTGAAKRGTTYGATYNTGGCTCAG) and (GTGACTGGAGTTCAGACGTGTGCTCTTCCGATCTACGTNTBACCGCDGCTGCTG)

I ran the cutadapt.sh file as follows: bash cutadapt.sh

## V45

Multiple adaptors were used in this case with variable lengths. This makes things a little more complicated to process

Adaptors used are:

- Forward: 515FP4-FwR1, 515FP3-FwR1, 515FP2-FwR1, 515FP1-FwR1 (ACACTCTTTCCCTACACGACGCTCTTCCGATCTCAAGTGCCAGCMGCCGCGGTAA , ACACTCTTTCCCTACACGACGCTCTTCCGATCTACGTGCCAGCMGCCGCGGTAA , ACACTCTTTCCCTACACGACGCTCTTCCGATCTTGTGCCAGCMGCCGCGGTAA , ACACTCTTTCCCTACACGACGCTCTTCCGATCTGTGCCAGCMGCCGCGGTAA)

- Reverse: 806RP4-RvR2 , 806RP3-RvR2 , 806RP2-RvR2 , 806RP1-RvR2 (GTGACTGGAGTTCAGACGTGTGCTCTTCCGATCTCATGGACTACHVGGGTWTCTAAT , GTGACTGGAGTTCAGACGTGTGCTCTTCCGATCTACGGACTACHVGGGTWTCTAAT , GTGACTGGAGTTCAGACGTGTGCTCTTCCGATCTTGGACTACHVGGGTWTCTAAT , GTGACTGGAGTTCAGACGTGTGCTCTTCCGATCTGGACTACHVGGGTWTCTAAT)

Based on the regions that are shared across the samples, and the fact that cutadapt will trim all the information past the region in the '3 primers, I have truncated the primers to the following:

- V45 adaptor 5' GTGCCAGCMGCCGCGGTAA

- V45 adaptor 3' GTGACTGGAGTTCAGACGTGTGCTCTTCCGATCT

I ran the cutadapt.sh file as follows: bash cutadapt.sh

# Next steps, each was run the same way through DADA2

Forward for V13 will be trimmed at 250 which is the estimation where the quality begins to crash

Reverse for V13 is much worse quality, which is expected, but this is the most errors I've seen. Will trim at 200 and will see if the two will align or not

## Turns out the fastq files that we got from them already had the adaptors pre-trimmed, or at least there is an error with the files after we remove the adaptors with cutadapt that they error out the file, so I ran the fastq files we got from them directly after renaming them in the normal Illumina convention ("_S#_L001_R1_001.fastq"). This is most likely the real scenario because V4-V5 has multiple kinds of primers so it would be very weird if they give you the unfiltered result

## After running Figaro for best parameters for trimming, this is the command we should run in DADA2

## Command used in DADA2 docker environment

## V13 (assumption in Figaro that V13 has a maximum of 490 bp in length)

library(dada2)

path <- "/data"

fnFs <- sort(list.files(path, pattern="_R1_001.fastq", full.names = TRUE))

fnRs <- sort(list.files(path, pattern="_R2_001.fastq", full.names = TRUE))

sample.names <- sapply(strsplit(basename(fnFs), "_"), `[`, 1)

filtFs <- file.path(path, "filtered", paste0(sample.names, "_F_filt.fastq.gz"))

filtRs <- file.path(path, "filtered", paste0(sample.names, "_R_filt.fastq.gz"))

names(filtFs) <- sample.names

names(filtRs) <- sample.names

# out <- filterAndTrim(fnFs, filtFs, fnRs, filtRs, truncLen=c(299,273),maxN=0, maxEE=c(5,8), truncQ=2, rm.phix=TRUE,compress=TRUE, multithread=TRUE)

out <- filterAndTrim(fnFs, filtFs, fnRs, filtRs, truncLen=c(270,250),maxN=0, maxEE=c(8,8), truncQ=2, rm.phix=TRUE,compress=TRUE, multithread=TRUE)

errF <- learnErrors(filtFs, multithread=TRUE)

errR <- learnErrors(filtRs, multithread=TRUE)

dadaFs <- dada(filtFs, err=errF, multithread=TRUE)

dadaRs <- dada(filtRs, err=errR, multithread=TRUE)

mergers <- mergePairs(dadaFs, filtFs, dadaRs, filtRs, verbose=TRUE)

seqtab <- makeSequenceTable(mergers)

write.table(seqtab,"/data/seqtableV13.tsv")

seqtab.nochim <- removeBimeraDenovo(seqtab, method="consensus", multithread=TRUE, verbose=TRUE)

sum(seqtab.nochim)/sum(seqtab)

# Then I took the output file, took all the sequences. Made them into a separate text file. Split them to 1 sequence at a time (issues with the computer not having enough resources to assign taxonomy) through the following shell command: split -l 1 v13_sequences.txt. Next I assigned taxonomy to each one separately

taxa <- assignTaxonomy(, "data/silva_nr99_v138.1_train_set.fa.gz",multithread=TRUE)

## V45 (assumption in Figaro that V13 has a maximum of 390 bp in length)

library(dada2)

path <- "/data"

fnFs <- sort(list.files(path, pattern="_R1_001.fastq", full.names = TRUE))

fnRs <- sort(list.files(path, pattern="_R2_001.fastq", full.names = TRUE))

sample.names <- sapply(strsplit(basename(fnFs), "_"), `[`, 1)

filtFs <- file.path(path, "filtered", paste0(sample.names, "_F_filt.fastq.gz"))

filtRs <- file.path(path, "filtered", paste0(sample.names, "_R_filt.fastq.gz"))

names(filtFs) <- sample.names

names(filtRs) <- sample.names

## Figaro determined this to be the best: c(178, 144) c(1, 1) 95.87 95.86624 300

out <- filterAndTrim(fnFs, filtFs, fnRs, filtRs, truncLen=c(178,144),maxN=0, maxEE=c(1,1), truncQ=2, rm.phix=TRUE,compress=TRUE, multithread=TRUE)

errF <- learnErrors(filtFs, multithread=TRUE)

errR <- learnErrors(filtRs, multithread=TRUE)

dadaFs <- dada(filtFs, err=errF, multithread=TRUE)

dadaRs <- dada(filtRs, err=errR, multithread=TRUE)

mergers <- mergePairs(dadaFs, filtFs, dadaRs, filtRs, verbose=TRUE)

seqtab <- makeSequenceTable(mergers)

write.table(seqtab,"/data/seqtable.tsv")

seqtab.nochim <- removeBimeraDenovo(seqtab, method="consensus", multithread=TRUE, verbose=TRUE)

sum(seqtab.nochim)/sum(seqtab)
