# Supplementary material for: Pre-treatment oral microbiome analysis and salivary Stephan curve kinetics in white spot lesion development in orthodontic patients wearing fixed appliances. A pilot study
Source: BMC Oral Health. 2023 Apr 24;23:239. doi: 10.1186/s12903-023-02917-z (PMC10127078; doi:10.1186/s12903-023-02917-z)
Supplement: Supplementary file 4 — Supplementary Material 4 [file 12903_2023_2917_MOESM4_ESM.docx]

**Supplementary File 2: libraries**

Hide

library(vegan)

library(tidyverse)

library(ggvegan)

library(gginnards)

library(ggrepel)

Load the OTU table in the format that Vegan approves of

Note that this rarefied document have had 1) OTUs condensed manually 2) mitochondria and chlorophylls removed

Hide

OTUtable = read_tsv("/Volumes/GoogleDrive/My Drive/Lab/saliva_stephans_curve/trial2/whitespots_rarefied_4931.tsv", col_names = TRUE, skip=1, show_col_types = FALSE) %>% as.data.frame(.)

titles = OTUtable[,1]

rownames(OTUtable) = titles

OTUtable[,1] = NULL

OTUtable = t(OTUtable)

head(OTUtable)

Hide

map = read_tsv("/Volumes/GoogleDrive/My Drive/Lab/saliva_stephans_curve/trial1/map/whitespots_rarefied.txt") %>% as.data.frame(.)

titles = map[,1]

rownames(map) = titles

map[,1] = NULL

map[,9] = NULL # Removes the color columns

map[,9] = NULL # Removes the color columns

map[,2] = NULL # Removes the sex column

nams=names(map)

head(map)

The following steps have been modified from the following tutorial (<https://rfunctions.blogspot.com/2016/11/canonical-correspondence-analysis-cca.html>)

Hide

OTUlog = decostand(OTUtable, "log")

ccamodel1 = cca(OTUlog ~ Disease + Age + Time0 + Time5 + Time15 + Time30 + Time45 , map)

ccamodel0 = cca(OTUlog ~ 1, map)

mod = step(ccamodel1, scope=list(

lower=formula(ccamodel0),

upper=formula(ccamodel1)),

direction="both") #AIC=125.59

mod_standard = step(ccamodel0,scope=formula(ccamodel1),test="perm") #AIC=125.59

# So basically nothing is really fitting a CCA model

I think its time to remove the variables that are no longer needed

Let’s redo the analysis but this time with Time as a delta from Time0

Hide

map = read_tsv("/Volumes/GoogleDrive/My Drive/Lab/saliva_stephans_curve/trial1/map/whitespots_rarefied_delta.txt") %>% as.data.frame(.)

titles = map[,1]

rownames(map) = titles

map[,1] = NULL

nams=names(map)

head(map)

Let’s do the same thing again now but with the new variables

Hide

OTUlog = decostand(OTUtable, "log")

ccamodel1 = cca(OTUlog ~ Disease + Age + Time0 + delta_time5 + delta_time15 +

delta_time30 + delta_time45, map)

ccamodel0 = cca(OTUlog ~ 1, map)

mod = ordistep(ccamodel1, scope=list(

lower=formula(ccamodel0),

upper=formula(ccamodel1)),

direction="both",

permutations=1000,

steps=1000) #In this case, we have delta_time15 being significantly different. What about interaction terms?

ccamodel3 = cca(OTUlog ~ Disease + Age + Time0 + delta_time5 + delta_time15 + delta_time30 + delta_time45 +

Disease*Age + Disease*Time0 + Disease*delta_time5 + Disease+delta_time15 + Disease*delta_time30 + Disease*delta_time45 + Age*Time0 + Age*delta_time5 + Age*delta_time15 + Age*delta_time30 + Age*delta_time45 + Time0*delta_time5 + Time0*delta_time15 + Time0*delta_time30 + Time0*delta_time45 + delta_time5*delta_time15 + delta_time5*delta_time30 + delta_time5*delta_time45 + delta_time15*delta_time30 + delta_time15*delta_time45 + delta_time30+delta_time45 , map)

mod2 = ordistep(ccamodel3, scope=list(

lower=formula(ccamodel0),

upper=formula(ccamodel3)),

direction="both",

permutations=1000,

steps=1000,

Pin=0.05,

Pout=0.06)

anova.cca(mod2,by = "margin")

print(mod2)

vif.cca(mod2)

From the looks of it, interaction terms are the most important components here. That is, Time0 with delta_time15, Time0 with delta_time30, and interaction between delta_time5 and delta_time45. But, the collinearity between Time0:delta_time15, Time0:delta_time30, delta_time5:delta_time45, delta_time30, and delta_time15 are very high. Will need to figure out what to do with that. Based on <https://sites.google.com/site/mb3gustame/warnings/warning-confounding-variables-and-multicollinearity>, removal of collinear terms is acceptable in exploratory analysis.

Hide

ccamodel4 = cca(OTUlog ~ Disease + Age + Time0 + delta_time5 + delta_time15 + delta_time30 + delta_time45 +

Disease*Age + Disease*Time0 + Disease*delta_time5 + Disease+delta_time15 + Disease*delta_time30 + Disease*delta_time45 + Age*Time0 + Age*delta_time5 + Age*delta_time15 + Age*delta_time30 + Age*delta_time45 + Time0*delta_time5 + Time0*delta_time30 + Time0*delta_time45 + delta_time5*delta_time15 + delta_time5*delta_time30 + delta_time5*delta_time45 + delta_time15*delta_time45 + delta_time30+delta_time45 , map) # Removed the most collinear variable, which is Time0:delta_time15, next step was remove delta_time15*delta_time30

mod3 = ordistep(ccamodel4, scope=list(

lower=formula(ccamodel0),

upper=formula(ccamodel3)),

direction="backward",

permutations=1000,

steps=1000,

Pin=0.05,

Pout=0.06)

anova.cca(mod3,by = "margin")

print(mod3)

vif.cca(mod3)

This model looks good, only 4 terms, and all are not collinear. Moving on

Time to graph them

<!-- rnb-text-end -->

<!-- rnb-chunk-begin -->

<!-- rnb-source-begin eyJkYXRhIjoiYGBgclxubXljY2EgPSBjY2EoZm9ybXVsYSA9IE9UVWxvZyB+IGRlbHRhX3RpbWU1ICsgZGVsdGFfdGltZTE1ICsgZGVsdGFfdGltZTQ1ICsgZGVsdGFfdGltZTUqZGVsdGFfdGltZTQ1LCBkYXRhID0gbWFwKVxuXG5hbm92YS5jY2EobXljY2EsYnk9XCJtYXJnaW5cIilcbnZpZi5jY2EobXljY2EpXG5cblxucGxvdChteWNjYSlcblxubXlnZyA9IGF1dG9wbG90KG15Y2NhKVxuXG5sYWJlbHMgPSBteWdnJGxheWVyc1tbMV1dJGRhdGEkTGFiZWxcbmNjYTEgPSBteWdnJGxheWVyc1tbMV1dJGRhdGEkQ0NBMVxuY2NhMiA9IG15Z2ckbGF5ZXJzW1sxXV0kZGF0YSRDQ0EyXG5sYWJlbHNfY29vcmRzID0gY2JpbmQobGFiZWxzLGNjYTEsY2NhMilcbnNhbXBsZU5hbWVzID0gcm93bmFtZXMoT1RVdGFibGUpXG5sYWJlbHNfY29vcmRzMiA9IHN1YnNldChsYWJlbHNfY29vcmRzLCAhbGFiZWxzICVpbiUgc2FtcGxlTmFtZXMgKSAlPiUgYXMuZGF0YS5mcmFtZSguKSAjIFJlbW92ZSB0aGUgc2FtcGxlIG5hbWVzIGFuZCBjb29yZGluYXRlc1xubGFiZWxzX2Nvb3JkczIkY2NhMSA9IGFzLm51bWVyaWMobGFiZWxzX2Nvb3JkczIkY2NhMSlcbmxhYmVsc19jb29yZHMyJGNjYTIgPSBhcy5udW1lcmljKGxhYmVsc19jb29yZHMyJGNjYTIpXG5cbm15Z2cyID0gbXlnZyArICBnZW9tX3RleHRfcmVwZWwoc2l6ZSA9IDUsIG1heC5vdmVybGFwcyA9IDEwMCxcbiAgYWVzKHggPSBsYWJlbHNfY29vcmRzMiRjY2ExLCB5ID0gbGFiZWxzX2Nvb3JkczIkY2NhMiwgbGFiZWwgPSBsYWJlbHNfY29vcmRzMiRsYWJlbHMgKSkgKyB0aGVtZV9saWdodCgpICsgZ2VvbV9qaXR0ZXIoKVxuXG5zdmcoXCJ+L0Rlc2t0b3AvdGVzdC5zdmdcIiwgaGVpZ2h0ID0gNDAsIHdpZHRoID0gNDApIFxubXlnZzJcbmRldi5vZmYoKVxuXG5teWdnMyA9IGRlbGV0ZV9sYXllcnMobXlnZzIsIFwiR2VvbVBvaW50XCIpXG5cbmBgYCJ9 -->

```r

mycca = cca(formula = OTUlog ~ delta_time5 + delta_time15 + delta_time45 + delta_time5*delta_time45, data = map)

anova.cca(mycca,by="margin")

vif.cca(mycca)

plot(mycca)

mygg = autoplot(mycca)

labels = mygg$layers[[1]]$data$Label

cca1 = mygg$layers[[1]]$data$CCA1

cca2 = mygg$layers[[1]]$data$CCA2

labels_coords = cbind(labels,cca1,cca2)

sampleNames = rownames(OTUtable)

labels_coords2 = subset(labels_coords, !labels %in% sampleNames ) %>% as.data.frame(.) # Remove the sample names and coordinates

labels_coords2$cca1 = as.numeric(labels_coords2$cca1)

labels_coords2$cca2 = as.numeric(labels_coords2$cca2)

mygg2 = mygg + geom_text_repel(size = 5, max.overlaps = 100,

aes(x = labels_coords2$cca1, y = labels_coords2$cca2, label = labels_coords2$labels )) + theme_light() + geom_jitter()

svg("~/Desktop/test.svg", height = 40, width = 40)

mygg2

dev.off()

mygg3 = delete_layers(mygg2, "GeomPoint")

Let’s see if bioenv has any solutions for what environmental variables are correlated with the distance matrix

Hide

# This time, let's try the full sequences, in PhILR format

OTUtable = read_tsv("/Volumes/GoogleDrive/My Drive/Lab/saliva_stephans_curve/trial2/white_spots_primer_averaged_with_code.tsv", col_names = TRUE, skip=1, show_col_types = FALSE) %>% as.data.frame(.)

titles = OTUtable[,1]

rownames(OTUtable) = titles

OTUtable[,1] = NULL

OTUtable = t(OTUtable)

suppressWarnings(suppressMessages(library(philr)))

suppressWarnings(suppressMessages(library(phyloseq)))

suppressWarnings(suppressMessages(library(ape)))

mytsv = otu_table(OTUtable, taxa_are_rows = FALSE)

mytree = suppressWarnings(dist(t(mytsv),method="euclidean")) %>% hclust(.,method="ward.D") %>% as.phylo(.)

phylo = suppressMessages(merge_phyloseq(mytsv,mytree))

isRooted = suppressWarnings(ape::is.rooted(phy_tree(phylo)))

isBinary = suppressWarnings(ape::is.binary(phy_tree(phylo)))

if(!isRooted){stop("Tree needs to be rooted.",call.=FALSE)} #if not rooted

if(!isBinary){phy_tree(phylo) = multi2di(phylo@phy_tree)} #if not binary tree

phy_tree(phylo) = ape::makeNodeLabel(phy_tree(phylo), method="number", prefix= 'n')

# Add 1 to avoid fractions on a zero denominator

data.no0 = transform_sample_counts(phylo, function(x) x+1)

phylot = merge_phyloseq(data.no0,phylo@phy_tree,phylo@sam_data,phylo@tax_table) # make your new GP phyloseq object based on the newly created matrix

myMatrix = phylot@otu_table@.Data

tree = phy_tree(phylot)

gp.philr = philr::philr(myMatrix, tree, part.weights='uniform', ilr.weights='uniform') %>% suppressMessages(.)

map_continuous = map

map_continuous[,1] = NULL

bioenv_results = bioenv(gp.philr ~ Age + Time0 + Time5 + Time15 + Time30 + Time45,map_continuous, metric = "euclidean",method="spearman",index="euclidean")

bioenv_results

So only Time5 is correlated with the matrix. Even still, very weak correlation.
